# Maternal stress and the early embryonic microenvironment: investigating long-term cortisol effects on bovine oviductal epithelial cells using air–liquid interface culture

**DOI:** 10.1186/s40104-024-01087-4

**Published:** 2024-10-03

**Authors:** Fiona Wahl, Jianchao Huo, Shuaizhi Du, Jennifer Schoen, Shuai Chen

**Affiliations:** 1 Department of Reproduction Biology, Leibniz Institute for Zoo and Wildlife Research (IZW), Alfred-Kowalke-Straße 17, 10315 Berlin, Germany; 2 Institute of Biotechnology, Technische Universität Berlin, Straße Des 17. Juni 135, 10623 Berlin, Germany; 3 Institute of Reproductive Biology, Research Institute for Farm Animal Biology (FBN), Wilhelm-Stahl-Allee 2, 18196 Dummerstorf, Germany

**Keywords:** Air–liquid interface, Bovine, Cortisol, Early embryonic microenvironment, Epithelial cells, Maternal stress, Oviduct

## Abstract

The oviduct epithelium is the initial maternal contact site for embryos after fertilization, offering the microenvironment before implantation. This early gestation period is particularly sensitive to stress, which can cause reduced fertility and reproductive disorders in mammals. Nevertheless, the local impact of elevated stress hormones on the oviduct epithelium has received limited attention to date, except for a few reports on polyovulatory species like mice and pigs. In this study, we focused on the effects of chronic maternal stress on cattle, given its association with infertility issues in this monoovulatory species. Bovine oviduct epithelial cells (BOEC) differentiated at the air–liquid interface (ALI) were stimulated with 250 nmol/L cortisol for 1 or 3 weeks. Subsequently, they were assessed for morphology, bioelectrical properties, and gene expression related to oviduct function, glucocorticoid pathway, cortisol metabolism, inflammation, and apoptosis. Results revealed adverse effects of cortisol on epithelium structure, featured by deciliation, vacuole formation, and multilayering. Additionally, cortisol exposure led to an increase in transepithelial potential difference, downregulated mRNA expression of the major glucocorticoid receptor (*NR3C1*), upregulated the expression of cortisol-responsive genes (*FKBP5*,* TSC22D3*), and significant downregulation of oviductal glycoprotein 1 (*OVGP1*) and steroid receptors *PGR* and *ESR1*. The systematic comparison to a similar experiment previously performed by us in porcine oviduct epithelial cells, indicated that bovine cultures were more susceptible to elevated cortisol levels than porcine. The distinct responses between both species are likely linked to their divergence in the cortisol-induced expression changes of *HSD11B2*, an enzyme controlling the cellular capacity to metabolise cortisol. These findings provide insights into the species-specific reactions and reproductive consequences triggered by maternal stress.

**Supplementary Information:**

The online version contains supplementary material available at 10.1186/s40104-024-01087-4.

## Introduction

Maternal stress is known to affect reproductive competence in mammals and can even lead to infertility [1, 2]. It is associated with impaired ovarian function [3, 4], compromised embryo development [3–7], foetal development retardation [8, 9], foetal losses [3, 9], reduced pregnancy rates [3, 10], and altered gestation length [11–14]. Numerous stressors were identified, including confinement/restraint [3, 5, 8, 15, 16], malnutrition [14], and psychological stress [1, 16, 17]. All these stressors lead to an increased release of glucocorticoids (GCs), such as cortisol, through stimulation of the hypothalamic–pituitary–adrenal (HPA) axis as a standard endocrine response to stress. Even though GC has a function in stress response, a controlled GC release by the HPA axis is also required to maintain reproductive function [18, 19]. The timing, concentration of GC, and glucocorticoid sensitivity of the tissue, therefore determine whether GC has a fertility-promoting or -inhibiting effect [18]. Severe or chronic stress can alter the normal pattern of cortisol excretion during the estrous cycle, disturbing the hormonal balance regulated by the hypothalamic–pituitary–gonadal (HPG) axis during crucial reproductive phases, consequently leading to reproductive dysfunction [20–23].

Next to the effect of stress-induced levels of GC on the HPG axis and the subsequent impairment of reproductive function caused by altered sex steroid patterns, GCs have also been shown to directly influence the female reproductive tract via local glucocorticoid receptors within cells [24]. The oviduct fulfils numerous functions, including facilitating the maturation and transport of gametes, posing as fertilisation site, supporting early embryonic development, all of which are necessary for a successful pregnancy outcome [25]. However, so far, it has received less attention in stress and GC signalling research than in the ovaries and uterus, although the early pregnancy stages occurring in the oviduct are considered the most susceptible to stress [26].

It was observed that restraint stress in mice, particularly during the period of embryo transport in the oviduct, resulted in elevated levels of GCs, decreased pregnancy rates, reduced litter sizes, and shorter pregnancy duration [15]. Exposure of porcine oviduct epithelium to elevated cortisol levels affected ciliated cell population, altered transepithelial bioelectric properties and changed the transcription profile [27, 28]. High GC levels have also been shown to increase necrosis and apoptosis in the oviduct epithelial cells (OEC) [15, 29–31], thereby impairing parthenogenic embryo development during co-culture [31]. These findings highlight the significance of the oviduct epithelium in stress-signal transduction affecting the preimplantation embryo [29]. Increased cortisol levels were further associated with an increased rate of polyspermy, suggesting that stress-hormones can hinder both fertilisation and embryo development in the oviduct [31].

Despite these recent advances, research on the effects of stress-induced GC levels on the oviduct has predominantly focused on polyovulatory, monogastric model species like mice and pigs. Cattle, on the contrary, are monoovulatory, polygastric ruminants, which are of crucial economic interest worldwide because of their importance to the meat and dairy industry. Infertility issues stemming from various stressors, including environmental, nutritional, or handling factors, pose a substantial threat to the industry’s productivity. Furthermore, cattle have been proposed as a model species sharing similar steroid and early embryonal development patterns with humans [32]. However, the bovine oviductal response to elevated cortisol levels has not been investigated so far, which would allow a better understanding of how stress influences the reproductive process in this species.

In the present study, we therefore conducted air–liquid interface (ALI) cultivation of bovine oviduct epithelial cells (BOEC), which allows recapitulation of in vivo functional properties and controlled long-term hormone stimulation. BOEC were stimulated with 250 nmol/L of cortisol, a level detected in cattle under severe stress [33, 34], for a duration of 3 weeks. Our primary focus was the chronic stress impact on the oviduct, as long-term approaches are strongly underrepresented. Morphological changes, transepithelial electrical properties, and gene expression were investigated and then compared to the results of a similar long-term cortisol stimulation study conducted by our group on porcine oviduct epithelial cells (POEC) [27].

## Materials and methods

### Media and reagents

HEPES, DPBS, penicillin/streptomycin, gentamycin, and amphotericin B were manufactured by Biowest (Nuaillé, France). Ethanol, BSA, formaldehyde, xylol, hemalum solution acid according to Mayer, eosin Y, and ROTI Histokitt II were obtained from Carl Roth (Karlsruhe, Germany). Other reagents were acquired from Sigma-Aldrich (St. Louis, MO, USA) unless stated otherwise.

Cultivation media are modifications of a protocol previously published by our group for ALI-BOEC cultivation [35]. The proliferation and differentiation media were formulated based on a basic medium consisting of DMEM/Ham’s F12 (Gibco, Fisher Scientific, Waltham, MA, USA), 15 mmol/L HEPES, 0.25 µg/mL amphotericin B, 100 U/mL penicillin, and 100 μg/mL streptomycin. The proliferation medium comprised the basic medium supplemented with 30 µg/mL bovine pituitary extract (Fisher Scientific, Waltham, MA, USA), 0.1 µg/mL cholera toxin, 25 ng/mL epidermal growth factor, 5% fetal bovine serum (Capricorn Scientific, Ebsdorfergrund, Germany), 10 µg/mL insulin, 0.05 µmol/L retinoic acid, and 5 µg/mL transferrin. The differentiation medium consisted of basic medium supplemented with 5% Nu-Serum (Fisher Scientific, Waltham, MA, USA) and 0.05 µmol/L retinoic acid to sustain the long-term differentiated status. With the addition of Nu-Serum to the differentiation medium, the final concentration of 17β-estradiol (E2) and progesterone (P4) are 149.50 pg/mL and 174,50 pg/mL, respectively.

### Tissue collection and cell isolation

Bovine oviduct tissue samples were obtained from slaughtered beef cows at a local slaughterhouse (Teterower Fleisch GmbH, Teterow, Germany) as by-products of the meat-production process. Hence, ethical approval was not applicable to this study.

Following collection, tissue samples were immediately washed twice with cold DPBS supplemented with 0.05 mg/mL gentamycin, 1 μg/mL amphotericin B, 100 U/mL penicillin, and 100 μg/mL streptomycin. Samples were transported to the lab on ice within 1 h, where the epithelial cells were isolated from both ipsilateral and contralateral oviducts according to the previously published protocol [35, 36]. The isolated cells were cryopreserved in liquid nitrogen for later use. Oviduct tissues collected from 3 animals were directly fixed in 4% paraformaldehyde overnight and then processed for histological analysis as described in the "Histology and histomorphometry" section below.

### ALI-BOEC culture

The ALI-BOEC culture was performed as recently described by our group [35, 36] with minor modifications. BOEC (*n* = 7 animals) were plated onto 0.4 µm pore size translucent 12-well inserts (Sarstedt, Nümbrecht, Germany). We seeded 3 inserts per animal and treatment group (control and cortisol), 1 for histology, 1 for RNA isolation and 1 as back-up. All inserts were pre-coated with 300 μL of bovine collagen IV solution (60 µg/mL) (Yo Protein, Ronninge, Sweden), and the seeding density was set at 6 × 10^5^ cells per insert. The proliferation phase was from d 0 to 6, with 1.5 mL of proliferation medium in the basolateral compartment and 400 µL in the apical compartment (liquid–liquid interface cultivation). From d 7 onward, cells were subjected to the ALI cultivation. This involved the application of differentiation medium to the basolateral compartment and the removal of the apical medium. All cultures were maintained in an incubator (Memmert, Schwabach, Germany) with 95% humidity at 37 °C, 5% CO_2_, and 18% O_2_, and medium was refreshed twice a week. After a total culture period of 4 weeks, cells were ready for the 3-week cortisol stimulation experiment (refer to the following section “Cortisol preparation and stimulation scheme”).

For the 1-week cortisol stimulation experiment, BOEC from a single animal (animal 2) were seeded onto 12-well inserts (11 replicates/condition) and cultured for a total of 4 weeks, following the procedure mentioned above.

### Cortisol preparation and stimulation scheme

Cortisol preparation and stimulation were performed following our previously reported procedure [27]. We selected a cortisol concentration of 250 nmol/L (90.63 ng/mL) for the stimulation, which reflects a severe stress-induced level in cattle [33, 34]. The final concentration of steroid solvent (ethanol) in the medium was 0.0009%. The solvent control was prepared accordingly.

**Fig. 1:**
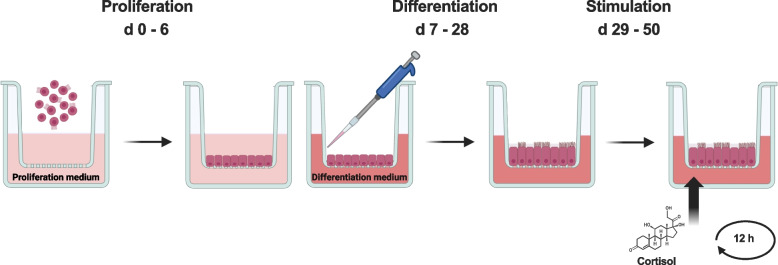
Schematic diagram depicting the 3-week cortisol stimulation procedure in ALI-BOEC. After 4 weeks of pre-culture, ALI-BOEC were stimulated with 250 nmol/L of cortisol from the basolateral compartment for 3 weeks. Medium refreshment was performed every 12 h to maintain a constant cortisol concentration. ALI, air–liquid interface; BOEC, bovine oviduct epithelial cells. Figure created with BioRender.com

*3-week cortisol stimulation*: following the 4-week cultivation period, the ALI-BOEC cultures were stimulated with cortisol for 3 weeks to simulate chronic stress in cows, as illustrated in the scheme (Fig. 1). The cultures were treated with 1.5 mL of differentiation medium containing 250 nmol/L cortisol from the basolateral compartment. To maintain a constant cortisol level, the medium was replaced every 12 h. This scheme for medium change was also applied to the control group.

*1-week cortisol stimulation*: to gain an insight into the mid-term effects of cortisol, the same cortisol stimulation procedure was conducted for 7 d.

### Transepithelial electrical resistance (TEER)/transepithelial potential difference (TEPD) measurements

At the end of the cortisol stimulation, transepithelial electrical resistance (TEER) and transepithelial potential difference (TEPD) measurements were performed in all inserts to assess epithelial barrier function and transepithelial ionic transport. These measurements were performed using an EVOM3-STX2-PLUS system (WPI, Sarasota, FL, USA) in accordance with the manufacturer’s instructions.

### Histology and histomorphometry

Oviduct tissue (ampulla region) from 3 animals, along with ALI-BOEC cultures, were processed for histological analysis to examine their morphology, cell composition, and differentiation status. After the TEER/TEPD measurement, we randomly selected 1 insert/animal/condition from the 3-week stimulation experiment (*n* = 7 animals) and 3 inserts/condition from the 1-week stimulation experiment for histological fixation. The cultures were washed with pre-warmed DPBS from both sides and then fixed in freshly prepared Bouin’s solution for 2 h. Next, the membrane was removed from the inserts and processed for histological fixation, dehydration, and embedding, as previously detailed by our group [36]. The oviduct tissues were fixed in 4% paraformaldehyde overnight, then dehydrated and embedded alongside the ALI-BOEC cultures. Afterwards, 3 µm sections were prepared from both paraplast-embedded oviduct tissues and BOEC cultures for immunohistochemistry (IHC) and hematoxylin–eosin (HE) staining. The slides were imaged using an Olympus IX81 microscope (Olympus, Germany) coupled with the CellSens imaging software. In terms of HE staining of BOEC, five images per insert were captured for morphology scoring.

To quantitatively analyse the morphology of the BOEC layers and facilitate morphological comparison among animals, a morphological scoring system was implemented. The HE stained epithelia were scored using the criteria displayed in Table 1, which include ciliation, polarity, confluency, the presence of vacuoles, homogeneity, and monolayer formation. The maximum score is 10, corresponding to a fully differentiated epithelium consisting of a ciliated, well-polarized, and homogenous epithelial monolayer without vacuoles.

**Table 1:** Criteria for morphological scoring of ALI-BOEC cultures

	**0 Point**	**1 Point**	**2 Points**
Ciliation	No cilia	Yes, but rare	Moderate or dense
Cell polarity	Flat	Partly columnar	Columnar
Confluency	Not confluent	Partly confluent	Full confluency
Appearance of vacuoles	In > 20% cells	In < 20% of cells	None
Homogeneity	Inhomogeneous	Homogeneous	-
Monolayer formation	Partly multilayered	Monolayer	-

### Immunohistochemistry

Immunolocalization of oviduct marker proteins was performed on both oviduct tissues (*n* = 3 animals) and control BOEC cultures (*n* = 3 animals) for comparison of cellular traits in vivo and in vitro. The sections underwent deparaffinization and rehydration, followed by antigen retrieval, which was performed by microwaving in 10 mmol/L sodium citrate buffer (pH 6.0) for 8 min. Endogenous peroxidase activity was quenched by incubating in 3% H_2_O_2_ in methanol for 10 min. To minimize non-specific binding, sections were blocked in a buffer containing 5% BSA and 2% horse serum (MP-7402, Vector Laboratories Inc, Burlingame, USA) for 1 h. Afterwards, the sections were incubated overnight at 4 °C with either a polyclonal rabbit anti-OVGP1 primary antibody (ab118590, Abcam, Cambridge, UK, 1:400, RRID:AB_10898500), or for 1.5 h at room temperature with a monoclonal mouse anti-acetylated tubulin primary antibody (T7451, Sigma-Aldrich, St. Louis, USA, 1:4,000, RRID: AB_609894). Sections that did not receive the primary antibody served as negative controls. After labelling with primary antibody, the sections were incubated for 1 h at room temperature with peroxidase-conjugated anti-rabbit or anti-mouse IgG secondary antibody (MP-6401, MP-7402, Vector Laboratories Inc, Burlingame, USA). The immunoperoxidase color reaction was visualized using a diaminobenzidine substrate chromogen solution (Dako, Carpinteria, CA, USA) and slides were counterstained with hematoxylin.

### Gene expression analysis

For the 3-week stimulation experiment, one 12-well insert per animal and treatment group was used for RT-qPCR analysis, as recently reported by Chen et al. [37]. In summary, total RNA from the ALI-BOEC cultures (*n* = 7 animals) was extracted using the NucleoSpin RNA kit (Macherey–Nagel, Dueren, Germany) and quantified using NanoDrop 2000c (Fisher Scientific, Waltham, MA, USA). Afterwards, cDNA was synthesized from 1 µg of total mRNA by RevertAid reverse transcriptase (Fisher Scientific, Waltham, MA, USA). Each qPCR analysis was performed in duplicates using SensiFast SYBR No-ROX Mix reagents (Meridian Bioscience, Cincinnati, OH, USA) and a CFX96 Touch-Real-Time PCR Detection System (Bio-Rad Laboratories Inc, Hercules, CA, USA). Replicates valid for analysis exhibited ≤ 0.4 standard deviation between single measurements. The threshold cycle (C_T_) value was automatically computed for each reaction using the analysis software LightCycler 96 (Roche, Basel, Switzerland). All primers exhibited an amplification efficiency of ≥ 90% and their details are listed in Additional file 1: Table S1. The specificity of the qPCR reactions was determined through melting curve analysis. The C_T_ values were then converted into relative quantities in comparison to one randomly selected control sample using the 2^−ΔΔCT^ method and corrected by the corresponding primer efficiency. The stability of five reference genes, including beta-actin (*ACTB*), succinate dehydrogenase complex flavoprotein subunit A (*SDHA*), glyceraldehyde-3-phosphate dehydrogenase (*GAPDH*), tyrosine 3-monooxygenase/tryptophan 5-monooxygenase activation protein zeta (*YWHAZ*), and transforming growth factor beta-stimulated clone 22 domain family member 2 (*TSC22D2*) were determined using the geNorm algorithm as described by Vandesompele et al. [38]. The normalization factor was generated based on the geometric mean of the two most stable endogenous reference genes (*YWHAZ* and *SDHA*).

### Western blot

ALI-BOEC cultures (*n* = 3 animals) from both control and cortisol-treated groups were lysed using ice-cold RIPA buffer (Cell Signaling Technology, Danvers, USA). The supernatant was collected after centrifugation at 14,000 × *g* for 30 min at 4 °C. Protein concentration was quantified using a Micro BCA Protein Assay Kit (ThermoFisher Scientific, Waltham, MA, USA). Subsequently, 10 µg of total protein from each sample was denatured at 95 °C for 5 min by mixing with Pierce Lane Marker Reducing Sample Buffer (ThermoFisher Scientific, Waltham, MA, USA). The Western blot procedure followed our recently published protocol [39]. Membranes were incubated overnight at 4 °C with polyclonal rabbit anti-OVGP1 primary antibody (ab118590, Abcam, Cambridge, UK, 1:1,500, RRID:AB_10898500). The secondary antibodies was HRP-conjugated goat anti-rabbit IgG antibody (7074S, Cell Signaling Technology, Danvers, USA, 1:2,000, RRID:AB_2099233). Chemiluminescence detection was performed using the ECL Prime Western Blotting Detection Reagent (GE Healthcare, Chicago, USA). The band quantification was conducted using ImageJ software. The region of interest (ROI) corresponding to the core non-glycosylated OVGP1 isoform (~ 60 kDa) was manually identified. The membrane image was converted to 8-bit format for uncalibrated optical density analysis. Background noise was removed using the light background method. Each band was individually selected and outlined with the rectangular ROI selection and “Gels” function. The peak areas of the resulting histograms were quantified, and data were recorded as arbitrary units.

### Statistical analysis

All data were statistically analysed using SPSS Statistics 29.0 (IBM, Armonk, NY, USA) for Windows. For the 3-week stimulation, data obtained from the TEER/TEPD measurements, histomorphometry, and gene expression analysis were analysed using a general linear model, with treatment as the main factor and animal as a random factor. For datasets where the residuals did not follow normal distribution or the variance of residuals is non-constant, the Wilcoxon rank sum test was applied to compare the cortisol treated group with control group. Data from the 1-week stimulation were analysed using Student’s *t*-test. A significance level of *P* < 0.05 was considered significant for all test values. Figures were generated and edited by GraphPad Prism 10 (GraphPad Software, San Diego, CA, USA) and BioRender unless stated otherwise.

## Results

### Differentiated ALI-BOEC cultures mimic the oviduct epithelium

**Fig. 2:**
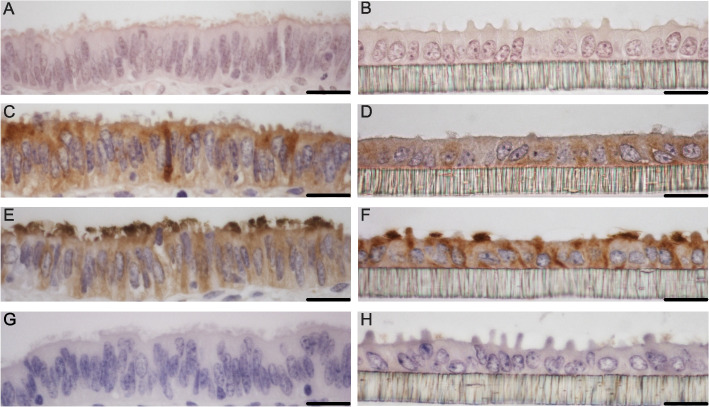
Long-term culture of ALI-BOEC recapitulates the morphology and properties of oviduct epithelium in vivo. **A**, **C**, **E**, **G** Bovine oviduct tissues (ampulla), *n* = 3 animals. **B**, **D**, **F**, **H** ALI-BOEC cultures from the control group of 3-week cortisol stimulation after 7 weeks of culture, *n* = 3 animals. **A** and **B** Representative HE-stained images; **C** and **D** immunohistochemical detection for OVGP1; **E** and **F** immunohistochemical detection for acetylated tubulin; **G** and **H** representative negative controls for the immunohistochemical staining. Scale bar = 20 µm. ALI, air–liquid interface; BOEC, bovine oviduct epithelial cells; OVGP1, oviduct glycoprotein 1

In this study, BOEC cultures were maintained in ALI culture for up to 7 weeks, during which they retained a well-differentiated status closely resembling in vivo oviduct tissue (Fig. 2A and B). The cultures formed a columnar monolayer composed of both ciliated and secretory cell populations, although the degree of polarization was somewhat less pronounced compared to natural oviduct tissue. Oviduct-specific glycoprotein 1 (OVGP1), a specific marker of oviductal epithelium, was expressed in the cytoplasm, as illustrated in Fig. 2C and D. Additionally, the presence of motile cilia on the apical surface of the epithelium was confirmed by strong positive staining for acetylated tubulin (Fig. 2E and F).

### Long-term cortisol stimulation impaired the morphology of ALI-BOEC

**Fig. 3:**
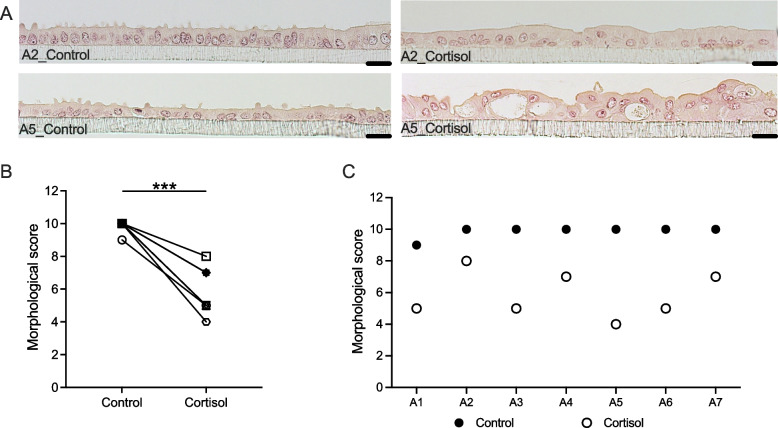
Morphological analysis of ALI-BOEC subjected to 3-week cortisol stimulation. **A** HE-stained images of ALI-BOEC cultures from animal 2 and animal 5 in both the control and 3-week cortisol-treated groups, taken at 200 × magnification, scale bar = 20 µm. **B** and **C** Changes in morphological score after 3 weeks of cortisol stimulation. *n* = 7 animals (A1–A7), representing animals 1 through 7. Asterisks indicate significance with ***, *P* < 0.001. ALI, air–liquid interface; BOEC, bovine oviduct epithelial cells; HE, hematoxylin–eosin staining

In the 3-week cortisol stimulation experiment, the control samples from all biological replicates demonstrated well-differentiated epithelial tissue formation (Fig. 3A). These samples showcased a polarised monolayer comprising both ciliated and secretory cells. Quantitative morphological scoring revealed that cultures from 6 out of 7 animals achieved the highest score of 10 points, while 1 animal (animal 1) yielded 9 points due to less extensive ciliation (Fig. 3C). Long-term exposure of ALI-BOEC to 250 nmol/L cortisol resulted in a considerable, albeit variable, decline in morphological quality across all animals (treatment factor: *P* < 0.001, animal factor: not significant (ns), Fig. 3B and C). Specifically, cultures derived from 4 animals (animals 1, 3, 5, 6) were significantly compromised by cortisol stimulation with a morphological score of ≤ 5; these epithelial cultures displayed increased presence of vacuoles, severe de-ciliation, reduced homogeneity, and partial multilayer formation following cortisol stimulation, as exemplified by animal 5 (Fig. 3A and C). 3 other animals (animals 2, 4, 7) depicted a less pronounced reduction in morphological quality following cortisol stimulation, achieving a moderate morphological score between 7 and 8 (Fig. 3C). These cells exhibited some vacuolisation along the epithelial layer, fewer cilia, and decreased uniformity compared to the control, as exemplified by animal 2, which received a morphological score of 8 (Fig. 3A).

### Long-term cortisol stimulation induced transepithelial bioelectric properties changes

**Fig. 4:**
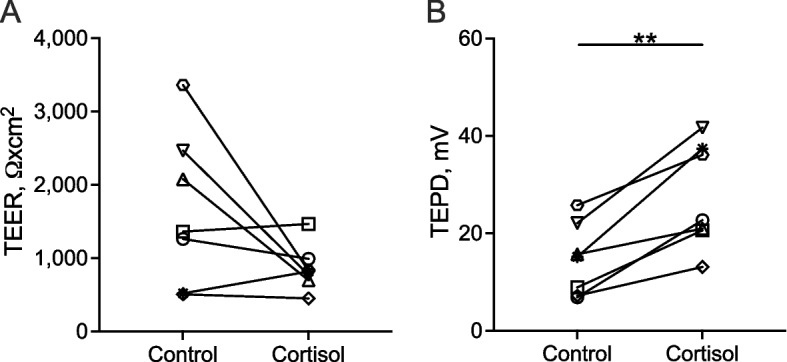
Transepithelial bioelectric properties of ALI-BOEC after long-term cortisol stimulation. **A** and **B** TEER and TEPD after 3-week treatment with 250 nmol/L cortisol, respectively. *n* = 7 animals, asterisks indicate significance with **, *P* < 0.01. ALI, air–liquid interface; BOEC, bovine oviduct epithelial cells; TEER, transepithelial electrical resistance; TEPD, transepithelial potential difference

To assess the epithelial bioelectric properties of ALI-BOEC in response to the 3-week cortisol stimulation, TEER and TEPD measurement was performed at the end of stimulation period. The TEER, associated with the epithelial barrier function, showed an average decrease of 862.2 ± 313.4 Ω×cm^2^ after cortisol stimulation, however, this change was not recognised as significant (Fig. 4A). Additionally, there is a parallel trend between the variations in TEER values and changes in morphological scores following 3-week cortisol stimulation (Additional file 2: Fig. S1). The TEPD quantifies the voltage or electrical potential between the apical (as reference) and basolateral sides of cells, reflecting the movement of ions across the epithelial layer. Following cortisol stimulation, there was a significant increase in voltage (treatment factor: *P* < 0.01, animal factor: *P* < 0.05, Fig. 4B). In the control group, TEPD ranged between 6.9 mV and 25.8 mV, with an average of 15.1 ± 7.5 mV. Conversely, cortisol-treated samples covered a range between 13.1 mV and 41.7 mV, averaging 27.5 ± 10.7 mV.

### Modifications in epithelial structure induced by 1-week cortisol stimulation

The impact on the epithelial structure in ALI-BOEC became noticeable already after 1 week of exposure to cortisol: signs of multilayering and non-homogeneity within certain segments of the epithelial layer were observed (Additional file 3: Fig. S2A and B). Morphological scoring revealed a mild drop in morphological quality (Additional file 3: Fig. S2C). Additionally, a significant decline in TEER (*P* < 0.01) and an increase in TEPD (*P* < 0.001, Additional file 3: Fig. S2D and E) was evident following 1-week cortisol stimulation. Notably, the shifts in TEER and TEPD values observed after 1 week of cortisol stimulation mirrored those after 3 weeks, affirming the regulatory effects of cortisol on epithelial bioelectrical properties in ALI-BOEC.

### 3-week cortisol stimulation affects gene expression in ALI-BOEC

#### Glucocorticoid signalling pathway

**Fig. 5:**
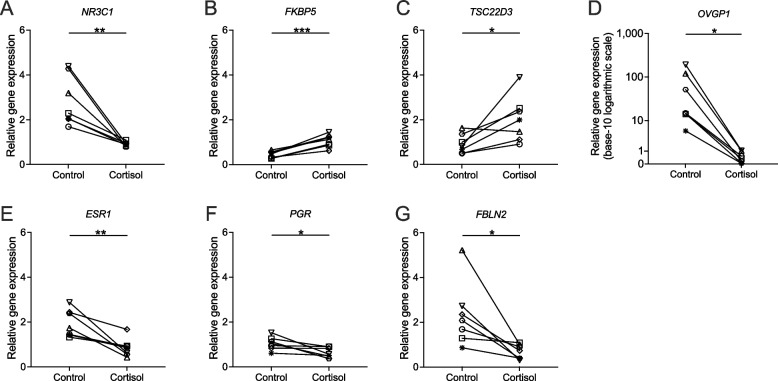
Relative expression of genes related to the glucocorticoid signalling pathway and oviductal functions in ALI-BOEC after 3-week cortisol stimulation. **D** The expression of *OVGP1* is depicted on a logarithmic scale chart with a base of 10, due to the substantial disparity in data between the cortisol-treated and control groups. Data are presented in connected dot plots. *n* = 7 animals, asterisks indicate significance with (***, *P* < 0.001; **, *P* < 0.01; *, *P* < 0.05). ALI, air–liquid interface; BOEC, bovine oviduct epithelial cells

We initially investigated the impact of prolonged cortisol stimulation on the expression of genes associated with the glucocorticoid pathway within ALI-BOEC. The mRNA expression of the glucocorticoid receptor, encoded by the nuclear receptor subfamily 3 group C member 1 (*NR3C1*), exhibited a significant downregulation (treatment factor: *P* < 0.01, animal factor: ns) after 3-week treatment with cortisol in all biological replicates (Fig. 5A). Moreover, the expression of the GR co-factor FKBP prolyl lsomerase 5 (*FKBP5*) and the cortisol-inducible gene encoding for the TSC22 domain family member 3 (*TSC22D3)* were both markedly upregulated by over two folds in comparison to the control (*FKBP5:* treatment factor: *P* < 0.001, animal factor: ns; *TSC22D3*: treatment factor: *P* < 0.05, animal factor: ns, Fig. 5B and C).

#### Selected oviduct-specific marker genes

After a 3-week stimulation with 250 nmol/L cortisol, the transcription of the classical oviduct marker, *OVGP1*, was significantly diminished (*P* < 0.05, Fig. 5D). Owing to a considerable disparity between the control and cortisol-treated groups, *OVGP1* expression is depicted on a base-10 logarithmic scale in Fig. 5D. Within the control group, a broad spectrum of expression levels was observed among the samples, reflecting substantial diversity across the animals. Nevertheless, the long-term cortisol treatment consistently and substantially suppressed *OVGP1* gene expression in all samples by 93% to 99%. It is to be noted that the *OVGP1* gene exhibited the most pronounced downregulation observed. The protein expression of OVGP1 was further quantified by Western blot in 3 animals (Additional file 4: Fig. S3A). A notable downregulation of the core non-glycosylated OVGP1 protein was observed in all cortisol-treated samples, consistent with the trend seen in gene expression (Additional file 4: Fig. S3B and C).

The expression of sex steroid receptors, estrogen receptor 1 (*ESR1*) and progesterone receptor (*PGR*), was assessed, given their pivotal roles in regulating oviduct epithelium functions across the estrous cycle. Following a 3-week cortisol treatment, both receptors experienced significant downregulation (*ESR1*: treatment factor: *P* < 0.01, animal factor: ns; *PGR*: treatment factor: *P* < 0.05, animal factor: ns, Fig. 5E and F). Additionally, the expression of the extracellular matrix protein fibulin 2 (*FBLN2*) was significantly decreased compared to the control group (treatment factor: *P* < 0.05, animal factor: ns, Fig. 5G).

#### Cortisol metabolism

**Fig. 6:**
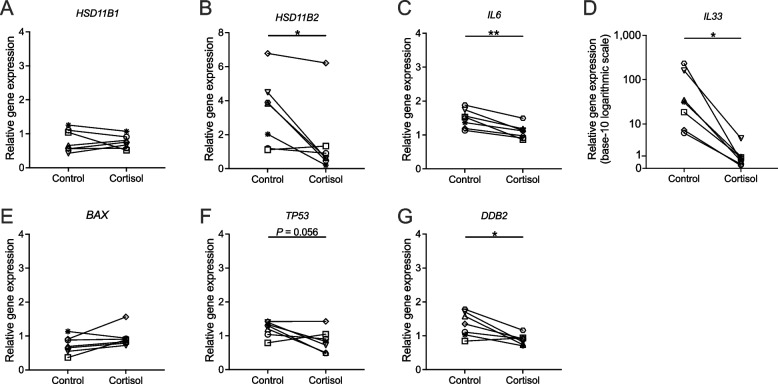
Relative expression of genes related to cortisol metabolism, inflammation, and apoptosis in ALI-BOEC after 3-week cortisol stimulation. **D** The expression of *IL33* is depicted on a logarithmic scale chart with a base of 10, due to the broad range of data points. Date are presented in connected dot plots. *n* = 7 animals, asterisks indicate significance with (**, *P* < 0.01; *, *P* < 0.05). ALI, air–liquid interface; BOEC, bovine oviduct epithelial cells

The cortisol metabolism involves two key enzymes: hydroxysteroid 11-beta dehydrogenase 1 (HSD11B1) catalyzes the conversion of inactive cortisone into active cortisol, while hydroxysteroid 11-beta dehydrogenase 2 (HSD11B2) reverses cortisol to cortisone. Upon 3-week cortisol stimulation in ALI-BOEC, there were no significant changes in the expression of *HSD11B1* (Fig. 6A), while *HSD11B2* expression exhibited a significant downregulation (treatment factor: *P* < 0.05, animal factor: *P* < 0.05, Fig. 6B). Upon individual examination of *HSD11B2* expression in each animal, a consistent downregulation was observed in BOEC derived from all subjects, except for animal 2, which displayed a 20% upregulation. Notably, unlike the other animals, animal 2 maintained the highest morphological score of 8 in the cortisol-treated group following a 3-week stimulation (Fig. 3C).

#### Pro-inflammatory cytokine expression and apoptosis

The expression of two pro-inflammatory cytokines, interleukin 33 (*IL33*) and interleukin 6 (*IL6*), was investigated considering cortisol’s immunosuppressive effect. Samples treated with cortisol for 3 weeks exhibited significant downregulation for both genes (*IL6*: treatment factor: *P* < 0.01, animal factor: *P* < 0.05; *IL33*: *P* < 0.05, Fig. 6C and D). Particularly noteworthy is the considerably high degree of downregulation by 85%–99% for *IL33*, as presented on a base-10 logarithmic scale in Fig. 6D.

The expression of pro-apoptotic marker BCL2 associated X (*BAX*) was unaffected by prolonged cortisol exposure (Fig. 6E), while the tumour suppressor gene tumor protein 53 (*TP53*) exhibited a slight downregulation compared to the control, although not reaching statistical significance (treatment factor: *P* = 0.056, animal factor: ns, Fig. 6F). The damage specific DNA binding protein 2 *(DDB2)*, which plays a major role in promoting the apoptotic process, was significantly downregulated (treatment factor: *P* < 0.05, animal factor: ns) after long-term cortisol treatment (Fig. 6G).

#### Comparative analysis between bovine and porcine OEC models on their responses to 3-week cortisol stimulation

The comparison between bovine and porcine OEC models relies on data from this study and our recent report on an analogous long-term cortisol stimulation approach in POEC [27], and is summarized in Table 2. Cultures from both species exhibited aligned regulatory trends in the majority of functional aspects, including transepithelial bioelectrical properties (TEER and TEPD), as well as genes related to 3 categories (glucocorticoid signalling pathway, inflammation, and apoptosis), hinting at the ubiquitous effects of cortisol on the oviduct epithelium across species. However, in contrast to porcine OEC cultures, the morphology of bovine OEC cultures was severely impaired following prolonged exposure to cortisol, coupled with a significant reduction in the expression of key oviduct function genes *OVGP1 *and* ESR1*. Likewise, the cortisol-metabolizing enzyme-encoding gene, *HSD11B2*, responsible for reducing biologically active cortisol levels, exhibited an opposite regulatory direction between the two species: while an upregulation was observed in POEC, a significant downregulation was detected in ALI-BOEC.

**Table 2:** Comparative analysis on the effect of 3-week cortisol stimulation in porcine and bovine ALI-OEC models

**Parameters**		**Bovine**	**Porcine**^**1**^	**Consistency**
Morphology		Severely impacted	Not impacted	No
Bioelectric properties	TEER	→	→	Yes
	TEPD	↑**	↑*	Yes
Gene expression				
Oviduct function	***ESR1***	↓**	→	No
	***OVGP1***	↓*	→	No
	*PGR*	↓*	↓*	Yes
Glucocorticoid signalling pathway	*FKBP5*	↑***	↑*	Yes
	*NR3C1*	↓**	↓*	Yes
	*TSC22D3*	↑*	↑*	Yes
Cortisol metabolism	*HSD11B1*	→	→	Yes
	***HSD11B2***	↓*	↑*	No
Inflammation	*IL6*	↓**	↓*	Yes
Apoptosis	*BAX*	→	→	Yes
	*DDB2*	↓*	↓*	Yes
	*TP53*	↓ (*P* = 0.056)	↓*	Trend: Yes

^1^Data from our previous study by Du et al. [27]. Arrows indicate the direction of regulation: ↑ upregulation, ↓ downregulation, → no expression change. Asterisks indicate significance with (***, *P* < 0.001; **, *P* < 0.01; *, *P* < 0.05). *n* = 7 animals in the current (bovine) and *n* = 5 in the previous (porcine) study

## Discussion

Cattle are known to experience reproductive challenges under stress, impairing various aspects such as corpus luteum formation, fertilisation rate, embryo and sperm quality, and increasing pregnancy loss, all of which lead to significant economic losses [40]. While the molecular pathway of stress via the central HPA system is well known, the localised effect of cortisol within the oviduct remains underexplored, with limited research conducted in polyovulatory animals, including pigs and mice. It is noteworthy that the pre-implantation state of pregnancy, in which the oviduct plays a prominent role, is especially susceptible to stress [26]. Therefore, the presented study is the first investigation into the effects of chronic stress on bovine reproduction competence by using long-term cortisol exposure on ALI-cultured oviduct cells.

The prolonged cortisol stimulation in the bovine OEC model resulted in disorganization of the oviduct epithelium, contrasting with our previous observations in the porcine model [27]. The adverse effects on morphology in BOEC cultures varied among animals, ranging from mild to severe, and was marked by a loss of ciliation and an increase in cytoplasmic vacuoles observed in all animals. Reduction in ciliated cells would directly impair the transport of gametes and early embryos within the oviduct in vivo [41, 42]. This decrease might be attributed to the downregulation of *ESR1* caused by the chronic cortisol stimulation, as the estrogen signalling is known to promote ciliogenesis in the oviduct [28]*.* On the other hand, the increased areas of secretory cells mimicked the earliest precursor lesions of oviduct-derived high-grade serous ovarian cancer [43]. Furthermore, we noted a diminished expression trend of the tumor suppressor *TP53*, along with its downstream gene *DDB2* [44], crucial for DNA damage response, following a 3-week cortisol treatment. This finding aligns with a previous study on chronic restraint stress in mice, illustrating that elevated glucocorticoids mediate the attenuation of p53 function and promotes tumorigenesis [45]. More than 50% of the ALI-BOEC cultures additionally exhibited a loss of homogeneity and the development of multilayers, a phenomenon evident as early as 1 week after cortisol stimulation. The functionality of the oviduct tissue is known to rely on the monolayer structure, and disorganisation of the epithelial layer is associated with diseases such as cancer [46]. The formation of multilayers following cortisol exposure may be attributed to the downregulation of extracellular matrix components, such as *FBLN2*, which plays a crucial role in preserving the basement membrane integrity of epithelium, and its reduced expression is linked to abnormal cell growth [47].

The disruption of BOEC structure due to prolonged cortisol exposure was corroborated at the gene expression level. Critical genes associated with oviduct function, such as the sex steroid receptors *ESR1* and *PGR1*, alongside the oviduct marker *OVGP1*, exhibited significant suppression. It has long been known that E2 and P4 play decisive roles, via binding to their respective nuclear receptors, in regulating the architectural and functional aspects of the oviduct, both during simulated cycles [37] and during estrous cycle in vivo. The oviductal transcriptome is globally dependent on *ESR1* and *PGR*, each controlling thousands of genes [48]. The downregulation of *ESR1* and *PGR* in ALI-BOEC suggests an impairment of the E2 and P4 signalling pathways within the oviduct, thereby influencing numerous downstream genes, as exemplified by the steroid-responsive gene *OVGP1*. *OVGP1* is the most abundant glycoprotein secreted by the oviduct, playing pivotal roles in modulating gametes and embryos during early reproduction events [49]. Its substantial downregulation by over 90% indicates a significant impairment of oviductal epithelium function induced by prolonged cortisol stimulation. Compared to the porcine study [27], it is noteworthy that the pronounced downregulation of *OVGP1* and *ESR1* in response to prolonged cortisol stimulation was observed exclusively in the bovine model, which exhibited impaired epithelial structure. This collectively suggests that cattle may be more susceptible than pigs to the harmful effects of chronically elevated cortisol levels on their oviductal function.

Common effects of cortisol on the glucocorticoid signalling pathway were observed at the transcriptional level in BOEC cultures, consistent with findings in porcine studies [27, 28]. Notably, there was a significant decrease in the glucocorticoid receptor *NR3C1* expression and an upregulation of *FKBP5* in the presence of cortisol. This suggests a negative feedback mechanism reducing sensitivity to glucocorticoid signalling to prevent overstimulation. Despite the adverse morphological changes observed in cortisol-treated BOEC, the glucocorticoid signalling pathway remained functional, as indicated by the upregulation of the cortisol-inducible gene *TSC22D3*.

The effects of cortisol stimulation on the bioelectrical properties of the bovine OEC model were found to align with our prior findings in the porcine OEC model, suggesting a conserved impact across species. Specifically, the TEPD, a voltage parameter reflecting ion transport like sodium and chloride across the epithelium, exhibited a consistent upregulation following 1 and 3 weeks of cortisol stimulation in ALI-BOEC. Similarly, in ALI-POEC, we observed a corresponding increase in TEPD after 3 d and 3 weeks of cortisol treatment [27, 28]. This pronounced elevation in TEPD signifies alterations in the ion composition within the apical fluid of epithelium, which raises concerns regarding the potential negative impact of elevated cortisol levels on the balance of the oviduct micro-environment, potentially disrupting crucial early reproductive events in vivo*.* The TEER, an indicator of the integrity and tightness of the epithelial cell layer, was rather unaffected following 3-week cortisol stimulation in the ALI-BOEC. Although there was an overall trend of reduction in TEER, these changes were not deemed statistically significant, with variability observed among individual animals. Likewise, in ALI-POEC, the changes in TEER following 3-week stimulation were not statistically significant when compared to the control group [27]. Additionally, in ALI-BOEC, we noticed that when the epithelial morphology is severely disrupted, as indicated by a significant drop in the morphological score, the TEER was also substantially decreased, exceeding −1,381 Ω×cm^2^ (Additional file 2: Fig. S1). This suggests that the non-invasive monitoring of TEER could mirror the epithelial structure changes in the ALI-BOEC culture.

### Species-specific responses toward long-term elevated cortisol exposure

A comparative analysis between the stress response in BOEC, as analysed in this study, and POEC, previously investigated by our group [27], is possible due to commonalities in cultivation conditions (such as cortisol concentration, cultivation system, stimulation procedure). BOEC and POEC exhibit consistent responses regarding bioelectric properties and main functional categories of selected genes, including the glucocorticoid signalling pathway, inflammation, and apoptosis, towards long-term exposure to elevated cortisol levels induced by severe stress. Nevertheless, alongside these common cortisol-induced effects, species-specific differences are evident: impairment on the epithelium structure and expression of major oviduct genes were exclusively observed in the bovine model, not in the porcine model.

The action of cortisol on the cells depends on its availability, mediated by the 11β-HSD enzyme system. In the bovine model, while the expression of *HSD11B1*, responsible for converting cortisone into cortisol, was not affected, the expression of *HSD11B2*, responsible for converting cortisol into inactive cortisone and thereby reducing cortisol availability to GR, was markedly downregulated following the 3-week cortisol treatment. This could lead to excessive accumulation of cortisol in the cells, suggesting a limited capacity of the bovine OEC model to withstand exposure to elevated levels of cortisol for prolonged period, as illustrated in their disrupted morphology. Contrarily, the porcine model showed an upregulation of *HSD11B2* expression post-cortisol treatment and efficiently reduced the high cortisol level by metabolizing it into cortisone [27]. Accordingly, the epithelium structure remained unaffected in the porcine OEC model. Notably, animal 2 of BOEC culture, the sole biological replicate showing a slight upregulation of *HSD11B2* in response to cortisol, maintained an intact morphology with the highest morphological score of 8 in the cortisol-treated samples. Those observations underscored the crucial role of cortisol-metabolising enzyme in shaping the oviductal stress response across different organisms. In alignment with our findings, Gong et al. [50] reported species-specific glucocorticoid metabolism between mouse and pig oocytes, resulting in a distinct glucocorticoid sensitivity in each species.

### Study limitations

In this study, BOEC were sourced from bovine oviducts obtained as by-products from slaughterhouses. Information on donor animals’ breed, age, health status, and cycle stage, factors that profoundly influence cell populations and characteristics, is unclear and presents significant diversity. This inherent animal variability was evident in the large differences observed in TEER measurements within the control group. Additionally, significant animal effects were detected in the TEPD measurements and in certain gene expression responses of BOEC cultures to cortisol. In the porcine study, cells also originated from slaughterhouse by-products. However, almost exclusively 6-month-old, pre-pubertal animals are used, so that the effects of cortisol are very consistent in the different biological POEC replicates. Sex-steroids are another factor which potentially interacts with cortisol in the stress responses of the oviduct epithelium [28]. Under the in vivo scenario, the levels of sex steroids fluctuate dynamically during the bovine estrous cycle. The differentiation medium used for stimulation contained 149.50 pg/mL of E2 and 174.50 pg/mL of P4, which provides some degree of sex-steroid support and allows for a more relevant examination of cortisol's actions. However, the model lacks the cyclic variations and precise hormonal regulation typically observed in vivo. Incorporating these elements will be crucial in future studies to enhance the physiological relevance of cortisol’s impact. The gene expression results need to be interpreted carefully, as transcriptional changes may not always comply with protein expression. Further investigations into the protein level and the enzyme activity of the glucocorticoid metabolizing enzymes, will facilitate corroboration of the findings in the future.

## Conclusion

To the best of our understanding, our study represents the first investigation into the local impact of severe chronic stress on the oviduct of monoovulatory bovines. Exposure of BOEC to elevated cortisol levels reflecting severe stress over a prolonged period induced pathological changes within the bovine oviduct, as evidenced by disorganized epithelial structure, alteration in transepithelial bioelectrical properties, and deprivation of crucial genes for oviduct function. The comparison to an analogous study in pigs revealed a greater sensitivity of bovine OEC towards elevated cortisol levels than porcine OEC. The species-specific stress responses are likely attributed to the divergent expression changes of the cortisol-metabolising enzyme *HSD11B2*, which controls the availability of cortisol to GR within cells. To conclude, our study offered novel insights into the species-specific connections between maternal stress and impaired fertility in mammals.

## Supplementary Information

**Additional file 1: Table S1.** Detailed information on the primers used for the RT-qPCR analysis in ALI-BOEC. ALI, air–liquid interface; BOEC, bovine oviduct epithelial cells.

**Additional file 2: Fig. S1.** The fluctuations in TEER values and alterations in morphological scores of ALI-BOEC in response to 3-week cortisol stimulation. ALI, air–liquid interface; BOEC, bovine oviduct epithelial cells; TEER, transepithelial electrical resistance.

**Additional file 3: Fig. S2.** The morphology and transepithelial bioelectric properties of ALI-BOEC after 1-week cortisol stimulation. **A** and **B** Representative HE sections of ALI-BOEC in the control (**A**) and (**B**) cortisol treated group, scale bar = 20 μm. **C **Morphological scoring of ALI-BOEC in response to 1-week cortisol treatment, *n* = 1 animal, *n* = 3 technical replicates. **D** and **E** The shifts in TEER (**D**) and TEPD (**E**) in response to 1-week cortisol treatment, *n* = 11 technical replicates. Asterisks indicate significance with (***, *P* < 0.001; **, *P* < 0.01). ALI, air–liquid interface; BOEC, bovine oviduct epithelial cells; TEER, transepithelial electrical resistance; TEPD, transepithelial potential difference.

**Additional file 4: Fig. S3.** Western blot and immunodetection of OVGP1 in ALI-BOEC cultures following 3-week cortisol stimulation, *n* = 3 animals. **A** Immunodetection of OVGP1 with the core non-glycosylated form of OVGP1highlighted by the red rectangle. **B** Relative fold changes in OVGP1 mRNA expression. **C **Relative fold changes in OVGP1 protein abundance. In (**B**) and (**C**), the expression level in the control group for each animal is set to 1, and the expression in cortisol-treated samples is normalized to the corresponding control sample of the same animal. A1, A2, and A3 represent animals 1, 2, and 3, respectively. ALI, air–liquid interface; BOEC, bovine oviduct epithelial cells; OVGP1, oviduct glycoprotein 1.

## Data Availability

The datasets used and analysed during the current study are available from the corresponding author upon reasonable request.

## Abbreviations

ACTB: Beta-actin
ALI: Air-liquid interface
BAX: BCL2-associated X
BOEC: Bovine oviduct epithelial cells
DDB2: Damage-specific DNA binding protein 2
E2: 17β-Estradiol
ESR1: Estrogen receptor 1
FBLN2: Fibulin 2
FKBP5: FKBP prolyl lsomerase 5
GAPDH: Glyceraldehyde-3-phosphate dehydrogenase
GC(s): Glucocorticoid(s)
GR: Glucocorticoid receptor
HE: Haematoxylin-eosin
HPA: Hypothalamic–pituitary–adrenal
HPG: Hypothalamic–pituitary–gonadal
HSD11B1: Hydroxysteroid 11-beta dehydrogenase 1
HSD11B2: Hydroxysteroid 11-beta dehydrogenase 2
IL6: Interleukin 6
IL33: Interleukin 33
NR3C1: Nuclear receptor subfamily 3 group C member 1
OEC: Oviduct epithelial cells
P4: Progesterone
PGR: Progesterone receptor
POEC: Porcine oviduct epithelial cells
SDHA: Succinate dehydrogenase complex flavoprotein subunit A
TEER: Transepithelial electrical resistance
TEPD: Transepithelial potential difference
TP53: Tumor protein p53
TSC22D2: TSC22 domain family member 2
TSC22D3: TSC22 domain family member 3
YWHAZ: Tyrosine 3-monooxygenase/tryptophan 5-monooxygenase activation protein zeta
