# Supplementary figures and images for: Maternal stress and the early embryonic microenvironment: investigating long-term cortisol effects on bovine oviductal epithelial cells using air–liquid interface culture
Source: J Anim Sci Biotechnol. 2024 Oct 3;15:129. doi: 10.1186/s40104-024-01087-4 (PMC11447938; doi:10.1186/s40104-024-01087-4)

### 3-week cortisol stimulation in ALI-BOEC

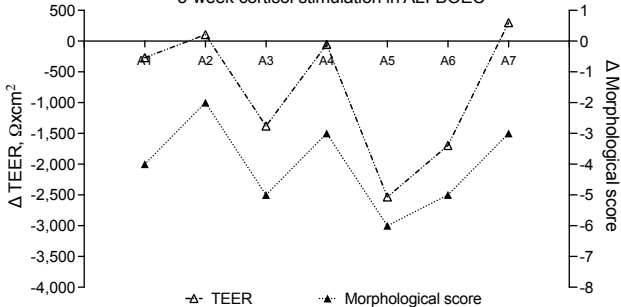

Supplement: Supplementary file 2 — Additional file 2: Fig. S1. The fluctuations in TEER values and alterations in morphological scores of ALI-BOEC in response to 3-week cortisol stimulation. ALI, air–liquid interface; BOEC, bovine oviduct epithelial cells; TEER, transepithelial electrical resistance. [file 40104_2024_1087_MOESM2_ESM.pdf]

**A**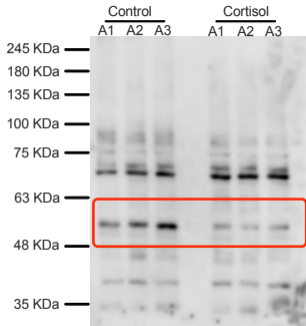**B**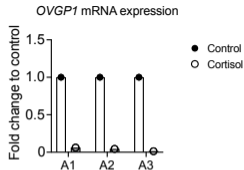**C**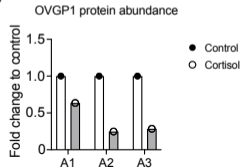

Supplement: Supplementary file 4 — Additional file 4: Fig. S3. Western blot and immunodetection of OVGP1 in ALI-BOEC cultures following 3-week cortisol stimulation, n = 3 animals. A Immunodetection of OVGP1 with the core non-glycosylated form of OVGP1highlighted by the red rectangle. B Relative fold changes in OVGP1 mRNA expression. C Relative fold changes in OVGP1 protein abundance. In (B) and (C), the expression level in the control group for each animal is set to 1, and the expression in cortisol-treated samples is normalized to the corresponding control sample of the same animal. A1, A2, and A3 represent animals 1, 2, and 3, respectively. ALI, air–liquid interface; BOEC, bovine oviduct epithelial cells; OVGP1, oviduct glycoprotein 1. [file 40104_2024_1087_MOESM4_ESM.pdf]
