# Supplementary material for: Maternal stress and the early embryonic microenvironment: investigating long-term cortisol effects on bovine oviductal epithelial cells using air–liquid interface culture
Source: J Anim Sci Biotechnol. 2024 Oct 3;15:129. doi: 10.1186/s40104-024-01087-4 (PMC11447938; doi:10.1186/s40104-024-01087-4)
Supplement: Supplementary file 3 — Additional file 3: Fig. S2. The morphology and transepithelial bioelectric properties of ALI-BOEC after 1-week cortisol stimulation. A and B Representative HE sections of ALI-BOEC in the control (A) and (B) cortisol treated group, scale bar = 20 μm. C Morphological scoring of ALI-BOEC in response to 1-week cortisol treatment, n = 1 animal, n = 3 technical replicates. D and E The shifts in TEER (D) and TEPD (E) in response to 1-week cortisol treatment, n = 11 technical replicates. Asterisks indicate significance with (***, P < 0.001; **, P < 0.01). ALI, air–liquid interface; BOEC, bovine oviduct epithelial cells; TEER, transepithelial electrical resistance; TEPD, transepithelial potential difference. [file 40104_2024_1087_MOESM3_ESM.pdf]

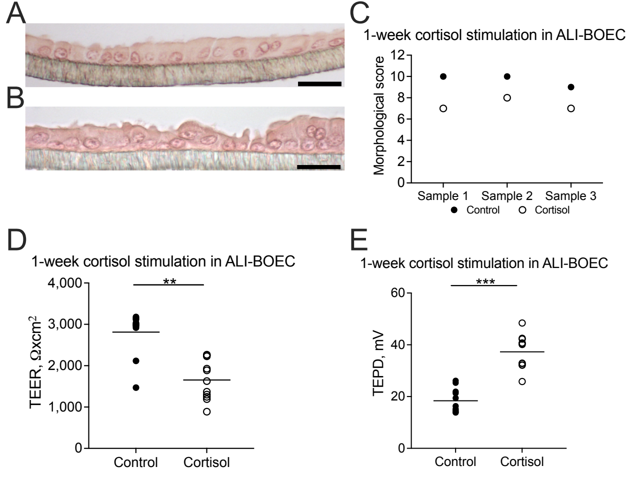
A
B
C
1-week cortisol stimulation in ALI-BOEC
Morphological score
12
10
8
6
4
2
0
Sample 1
Sample 2
Sample 3
Control
Cortisol
D
1-week cortisol stimulation in ALI-BOEC
**
TEER, Ωxcm2
4,000
3,000
2,000
1,000
0
Control
Cortisol
E
1-week cortisol stimulation in ALI-BOEC
***
TEPD, mV
60
40
20
0
Control
Cortisol
